# Protective effects of curcumin co-treatment in rats with establishing chronic variable stress on testis and reproductive hormones

**Published:** 2017-07

**Authors:** Masoomeh Mohamadpour, Ali Noorafshan, Saied Karbalay-Doust, Tahereh Talaei-Khozani, Elham Aliabadi

**Affiliations:** 1 *Histomorphometry and Stereology Research Center, Shiraz University of Medical Sciences, Shiraz, Iran.*; 2 *Anatomy Department, School of Medicine, Shiraz University of Medical Sciences, Shiraz, Iran.*

**Keywords:** Testosterone, Follicle stimulating hormone, Luteinizing hormone, CVS, Curcumin

## Abstract

**Background:**

Protracted and repeated exposure to chronic variable stress (CVS) may lead to reproductive dysfunction. It is a basic cause of male infertility. Curcumin (CUR) is an active fraction of turmeric that used in traditional Chinese medicine. CUR represents various pharmacological activities.

**Objective:**

The purpose of this study was to determining the effects of CUR on testis and testosterone, follicle stimulating hormone (FSH) and luteinizing hormone (LH) in rats with establishing chronic variable stress.

**Materials and Methods:**

Twenty-one adult male Sprague-Dawley rats were divided into three groups: 1) control, 2) CVS and 3) CVS+ CUR (100 mg/kg/day dissolved in 0.5 mL of olive oil). All of the animals in control, CVS, and CVS+CUR groups were sacrificed after 15 days. Testosterone, FSH, LH, and testis damage were evaluated.

**Results:**

Significant changes in the normal range of testosterone, FSH, LH serum levels and seminiferous tubule apoptotic cells were detected in CVS group compared to the control rats (p=0.02). These parameters changed to a less extent in CVS+CUR animals compared to the CVS rats (p=0.02).

**Conclusion:**

Our findings propose that curcumin might have curative potential on the reproductive system function and its impairment. It’s regulated by stress and reproductive-related hormones.

## Introduction

Stress is defined as a basic or perceived threat to the homeostasis of an organism (1, 2). Chronic variable stress (CVS) is major problem of the modern life, which led to changes in endocrine and reproductive functions along with the central and peripheral systems. Stress can be a big stimulator and sometimes as a strong factor in carrying out the activities (3). The CVS response also helps to preserve physical condition, disposition, efficiency, relationships and quality of life. Recently, based on some researches, CVS cause an interruption in the mammalian reproductive system including human (4, 5). Some studies have recommended that CVS might have adverse effects on the reproductive system. It has been shown that CVS could increase adrenal gland weight and also reduces pituitary, testis and seminal vesicle weight in rats that is associated with decreased spermatozoa count and cause male infertility (6, 7). In males, stress led to a significant increases in the levels of gonadotropin-releasing hormone, FSH and LH. CVS can also decrease testosterone level. Such effects might change sperm quality (8). Therefore; the first goal of the present study was to evaluate the effects of CVS on testosterone, LH, FSH and apoptosis of spermatogonia in rats (9). Several different approaches have been used for treatment of this impairment but the chemical treatment demonstrated a lot of side effects. So it seems essential to find a suitable therapy such as traditional medicine for neutralizing or decreasing these negative side effects.

Curcumin (CUR) that is a yellow color fraction in the rhizome of turmeric (*Curcuma longa*) has a broad collection of pharmacological and biological activities, including anti-inflammatory, anti-carcinogenesis and antioxidant activities. Recently, studies have shown that CUR has anti-apoptotic effects and act against toxic dexamethasone-induced apoptosis in rat thymocytes and chemotherapy-induced apoptosis in breast cancer cells (10, 11). CUR prevents ultraviolet irradiation-induced apoptotic changes, including loss of mitochondrial membrane potential, mitochondrial release of cytochrome C and decreases in reactive oxygen species. Some studies reveal benefit effects of CUR on testis damages (12-15). The second goal of the study was to introduce a protective agent, which could be available to the public without difficulty and be able to prevent the unpleasant effects of CVS on the testis. 

To reach these goals, this study was designed to answer the following questions: Does CVS change the testis apoptotic components quantitatively? Does CVS change testosterone, FSH and LH serum levels? Does CUR protect the rat testis exposed to CVS?

## Materials and methods

**Animals and experimental design**

Twenty-one adult male Sprague-Dawley rats weighing 220-250 gr (10 wk old) were randomly selected and acclimatized for two weeks prior to the experiment. Then they were divided into three groups. The first group (the control) not received treatment (n=7). While, the second group was exposed to CVS, accordance with the previous method with some modifications (n=7) (16, 17). The third group was exposed to CVS+CUR (100 mg/kg/day solved in 0.5 mL of olive oil) (n=7) (18). The administration was done by oral gavage. One day after the last treatment, animals were anesthetized with halothane and after collecting blood through the cardiac puncture they were euthanized by cervical dislocation. Testis from each animal was fixed in 10% formalin. The samples were embedded in paraffin, sectioned (5 μm) and stained with H&E for the morphometric study. Two blinded observers, analyzed the sections independently. Testis was also collected for apoptotic studies (19).

**CVS model**

The animals were exposed to CVS for 15 days (Table I). The following stressors were ordinary: cold restraint (1.5 hr), the inclination of home cages (3 hr), flashing light (1.5 hr), restraint (1.5 hr), isolation (24 hr), damp bedding (3 hr), water deprivation (24 hr), and no stressors. Restraint was performed by placing the rats in a 25×7 cm plastic cylinder with dumps for breathing. In addition, exposure to flashing light was accomplished by placing the rats in a 40×60×50 cm open area made of dark floor with a frontal glass wall. A 60 W lamp was flashed at a regularity of 60 flashes per minute (16). 

**Serum testosterone, LH and FSH levels**

At first, 5 mL blood samples were collected and centrifuged at 3000 rpm for 15 min. Then it was frozen at -20^o^C for 2 wk. The concentration of testosterone level was measured by Radioimmunoassay (RIA) kit Siemens ADVIA Centaur XP (Siemens; Erlangen, Germany), Serum FSH and LH were analyzed accordance with the method of Lin, Kawamura, Okamura and Mori respectively. All of the samples were analyzed at the same time (20).

**Morphometric estimation of the testis components**

To estimate the volume density “Vv (structure/testis)” of the seminiferous tubules (including the germinal epithelium and lumen) and interstitial tissue of the testis, the point-counting method was applied on the 5 µm sections at a final magnification of 39. Briefly, the total number of points hitting interstitial tissue, lumen or epithelium were counted and divided by a total number of points hitting the whole testis. The total volume of each structure was obtained by using the following formula (21): 

V (structure) = Vv(structure/testis)× V(testis) 

**Evaluation of germ cell apoptosis**

Testes were removed and fixed in 4% (v:v) paraformaldehyde for 16 hr at 4^o^C, then tissue processing (dehydration) was performed for 8 hr at 25^o^C, following by paraffin embedding. The specimens were subjected to TUNEL staining using an in situ Apoptosis Detection Kit (Catalogue No. 11684809910) according to the manufacturer's protocol. The TUNEL assay was carried out on paraffin-embedded sections of the testis. Briefly, sections were deparaffinized, cleared in xylene and rehydrated in graded concentrations of ethanol. The sections were digested with 20 mg/mL proteinase K for 15 min at room temperature. The sections were then washed and incubated with the TUNEL reaction mixture (enzyme solution and labeling solution) for 60 min at 37^o^C in a humidified atmosphere. Negative controls were processed according to the same protocol, except for the incubation with Terminal deoxynucleotidyl transferase. TUNEL-positive cell detection was based on dark labeling as intense as, or more intense than, that of apoptotic cells observed in the positive control slide labeled simultaneously. All labeled sections were viewed under the fluorescent microscope (Nickon, Eclipse, and E600). The number of TUNEL-positive cells in at least five cross sections of the tubules was counted. Immuno-staining intensity was estimated using a semi-quantitative score, HSCORE, method. The HSCORE was calculated for each section by application of the following algorithm: HSCORE= ΣPi (i+1), where i is the intensity of staining (0: no staining, 1: weak, 2: moderate, 3: strong) and Pi is the percentage of stained cells for each intensity (0-100%).

**Ethical consideration**

The experimental protocols were performed in the Shiraz University of Medical Science in Dec 2015-2016 according to the Shiraz University of Medical Science Ethics Guideline. The care and treatment of animals were also in accordance with ethical guidelines of the committee (approval No.94-7626). 

**Statistical analysis**

All data were presented in mean±SD. SPSS 16.0 (Statistical Package for the Social Sciences, version 16.0, SPSS Inc, Chicago, Illinois, USA) for windows was used to analyze the data. A nonparametric test weas used to analyze the data among the three groups. Dual comparisons between groups exhibiting significant values were evaluated with a Mann-Whitney U test. These differences were considered significant when probability was less than 0.05. 

## Results

**Serum testosterone, LH and FSH levels**

The serum testosterone, LH and FSH concentrations showed a significant decrease in the CVS animals, compared to the corresponding control rats (p=0.02). This parameter diminished to a lesser extent in the CVS+CUR treated animals compared to the CVS rats (p=0.02, Table II). 

**Morphometric estimation of the testis components**

In CVS group, the seminiferous tubules were located far from each other by increasing interstitial connective tissue. In CVS group, there was a reduction in spermatogenesis. The architecture of the testis was maintained, but the germinal epithelium showed inadequacy as well as marked degenerative changes. There was cell debris in the lumen of tubules as a result of the infusion of degenerated germ cells. Also, the interstitial spaces were increased. In CVS+CUR groups, we observed that the shape of seminiferous tubules were the same as those of control group. The germ cells in the tubules were arranged in an order like control animals. The degenerations seen in CVS group were disappeared (p=0.02).

**Evaluation of germ cell apoptosis**

TUNEL analysis showed that apoptosis was confined to the basal germ cells, indicating suppression of spermatogenesis. The number of TUNEL-positive cells in the testis markedly increased in CVS group (Figure I). Apoptotic cells were not detected in spermatids located close to the lumen. In the CVS animals, distended tubular lumen was seen due to reducing in the number of germ cells. The numbers of TUNEL-positive spermatogonia per seminiferous tubule of the CVS group increased compare to those of the control groups and it was found statistically significant decrease in CVS+CUR group as compared with the values of CVS group (p=0.02, Figure 2).

**Table I:** The protocol for induction of the chronic variable stress (CVS) in 15 days for the rat model

**Stressor applied**	**Days**
Cold restraint (1.5 hr)	1
Inclination of home cages (3 hr)	2
Flashing light (1.5 hr)	3
Restraint (1.5 hr)	4
Isolation (24 hr)	5
Damp bedding (3 hr)	6
Inclination of home cages (2 hr)	7
No stressor applied	8
Flashing light (1.5 hr)	9
Isolation (24 hr)	10
Water deprivation (24 hr)	11
Restraint (2 hr)	12
Damp bedding (2 hr)	13
Cold restraint (2 hr)	14
Inclination of home cages (3 hr)	15

**Table II:** Mean±SD serum testosterone (ng/ml), LH (ng/ml) and FSH (ng/ml) - in Control, Chronic variable stress (CVS) and Chronic variable stress+ Curcumin (CVS+CUR) groups.

**Groups**	**Testosterone**	**LH**	**FSH**
Control	1.7 ± 0.2	1.1 ± 0.2	6.1 ± 0.8
CVS	0.7 ± 0.1*	3.7 ± 0.3 *	13.3 ± 0.7 *
CVS+CUR	1.1 ± 0.2 *	2.1 ± 0.2 *	8.8 ± 0.5 *

CVS: Chronic variable stress

CUR: Curcumin

LH: Luteinizing hormone

FSH: Follicular stimulating hormone

* P=0.02 (Control *vs.*_._CVS) or ( CVS+CUR *vs.* CVS) evaluated with a Mann-Whitney U test

**Figure 1:**
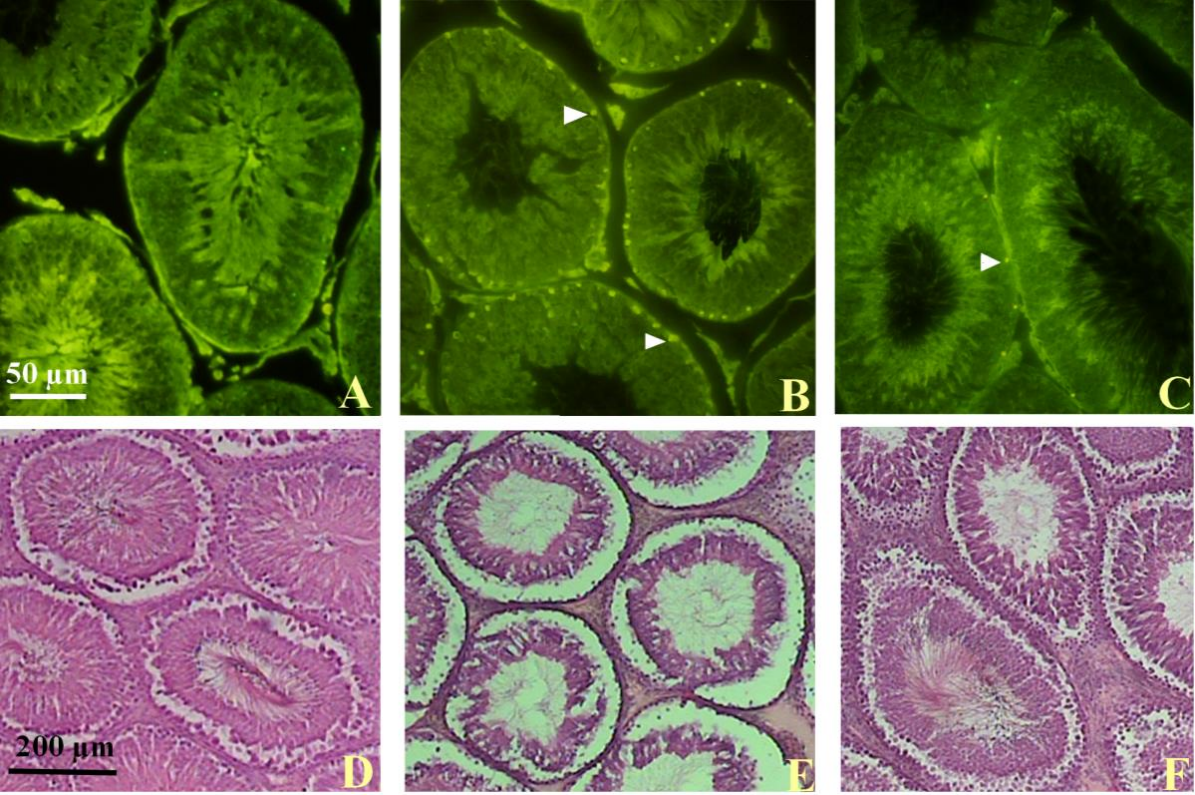
TUNEL and H & E staining of rat testis. Arrow heads indicate TUNEL-positive cells. A & D, Control, B & E CVS and C&F

CVS+CUR groups.(10X)

CVS: Chronic variable stress

CUR: Curcumin

**Figure 2:**
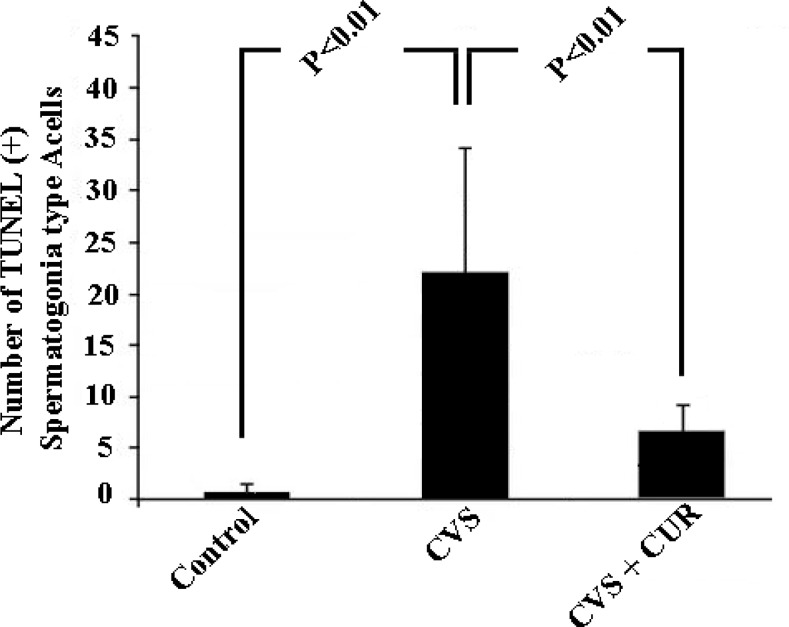
The mean and standard deviation of the percentage of TUNEL-positive spermatogonia per seminiferous tubules cross sections in control, Chronic variable stress (CVS) and Chronic variable stress+ Curcumin (CVS+CUR) animals (n= 7). **A.B** Different superscript letters indicate significant differences between.

CVS: Chronic variable stress

CUR: Curcumin

## Discussion

The data showed that CVS exerted a profound impact on hypophysis-gonad axis and as a result on the testis functions. Curcumin improve the advers effects of CVS. Previous studies confirmed that CVS disrupt normal homeostasis on a variety of body systems (22). CVS is known as an endocrine disruptor, it can influence the pituitary-testicular axis (22). CVS is also a major cause of male infertility following changes, such as decreased sex hormones, delayed ejaculation, low sexual desire, and low sperm quality (23). Accordance with other studies, this result showed that CVS significantly increased LH and FSH while decreased testosterone levels via the pituitary-testicular axis. In current study, it has been shown that levels of FSH and LH are elevated in exposed rats to CVS. FSH and LH respectively intervene in spermatogenesis and steroidogenesis by the affecting Sertoli and Leydig cells (24)‏. 

CVS led to decrease in the responsiveness of Leydig cells and serum testosterone and as a result, it led to an increase in LH level. The enhancement in serum FSH levels indicates a destruction of spermatogenesis in experimental rats and reflects the germ cell loss or damage to Sertoli cells that is because of disrupting regulation of FSH secretion (25). The reduction levels of serum testosterone with increased levels of FSH and LH in experimental rats also indicate an undamaged pituitary-testicular axis (26, 27). 

According to Chen and colleagues, reduction in testosterone level could be explained by an increase in cortisol concentration that might suppress Leydig cells through binding to the glucocorticoid receptors on the cells' surface. In our results, it was demonstrated that CUR can improve the reproductive hormone (testosterone, FSH, and LH) levels in the CVS rats. This might be due to the protective effect of CUR on Leydig cells. In support of our findings, previous researches approved the protective effects of CUR by using properties such as anti-apoptotic, anti-oxidative, and antigenotoxic (28). In addition, Abarikwu *et al*, Lonare *et al* and Smith *et al* reported that CUR inhibited cortisol secretion by suppressing adrenocorticotropic hormone and increasing mRNAs coding for steroid controlling proteins (29-31). 

Reserchers reports have indicated that serum testosterone level significantly increases after CUR treatment (32, 33). In this study, the size of the seminiferous tubules and spermatogenic cell in the CVS rats strongly reduced, also its epithelium severely impaired as seen in the morphology. In some studies stress is recognized as a strong mediator of apoptosis. In this process, mitochondria are known as an important factor. The mitochondrial dysfunction induced by oxidative stress, can lead to the release of cytochrome C and caspase activation that followed by cell death (34). 

In our study, testicular apoptosis in CVS rat showed possible role of the androgen reduction in germ cell apoptosis. Accordance with Yazawa *et al*, CVS intervenes in male reproductive action and increase apoptosis in the seminiferous tubules of the Rat (34). One of an important anti-apoptotic protein is B-cell lymphoma-2. CUR increases the expression of the B-cell lymphoma-2 protein and improves the spermatogenesis (35). CUR cause translocation of cytochrome C from mitochondria to cytosol and prevents mitochondria disruption (36). The current study showed that CVS significantly increase seminiferous tubule damages, which could be protected by CUR. Therefore, we suggest to investigate molecular pathways in future studies, especially in spermatogenesis cycle. 

## Conclusion

Exposure to 15 days of CVS altered the hormone levels and testis structures; and also increased apoptosis in rat germ cells. The findings also showed the possible protection role of CUR, as an easily available natural component, prevented testis damages after exposure to CVS. 

## Note

***This article extracted from the Ph.D. thesis. (Masoomeh Mohamadpour*** *)*
